# Goal-directed actions and habits in head-fixed mice

**DOI:** 10.3389/fnbeh.2026.1751553

**Published:** 2026-02-25

**Authors:** Logan M. Manusky, Lisa M. Green, Joshua A. Boquiren, Jade Baek, Marcus S. Bell, Kate M. Wassum, James M. Otis

**Affiliations:** 1 Department of Neuroscience, Medical University of South Carolina, Charleston, SC, United States; 2 Department of Psychology and Brain Research Institute, UCLA, Los Angeles, CA, United States; 3 Ralph H. Johnson VA Medical Center, Charleston, SC, United States; 4 Hollings Cancer Center, Medical University of South Carolina, Charleston, SC, United States

**Keywords:** behavioral neuroscience, calcium imaging, chemogenetics, dorsolateral striatum, goal-directed behavior, habitual behavior, striatum, two-photon

## Abstract

**Background:**

Behavioral control is fundamentally governed by a dynamic balance between flexible, goal-directed actions and efficient but highly automated habits. While the transition to habitual control can be adaptive, an imbalance favoring rigid habits over flexible control is thought to contribute significantly to the core pathology of many neuropsychiatric disorders. Despite their pervasiveness, the precise neural circuits that govern this critical balance remain poorly characterized. A major barrier to progress lies in the technical challenge of tracking single neurons in relevant circuits as goal-directed behavior becomes habitual.

**Methods:**

Here, we introduce and validate a novel head-fixed instrumental learning paradigm in mice that enables the differentiation of goal-directed and habitual behavioral control. This model provides an unprecedented platform for high-resolution, longitudinal *in vivo* neural interrogation. To functionally validate the behavioral paradigm, we employed chemogenetic inhibition of the dorsolateral striatum (DLS) in limited-trained and overtrained mice.

**Results:**

Our findings demonstrate that mice rapidly acquire lever pressing, exhibiting robust active/inactive lever discrimination, and comparable initial learning between limited and overtrained cohorts. Crucially, while mice with limited training readily reduced their lever-press behavior when the resulting outcome was devalued or omitted, overtrained mice displayed characteristic insensitivity to outcome devaluation and omission. Furthermore, chemogenetic inhibition of the DLS in overtrained mice restored sensitivity to both devaluation and contingency reversal, blocking habit expression and preserving goal-directed control.

**Conclusion:**

These results establish a paradigm for studying both goal-directed actions and habits in head-fixed mice and validate the DLS as a key neuronal substrate as previously demonstrated in freely moving models. This paradigm represents a vital methodological advancement that overcomes the technical barriers to longitudinal, cellular-level interrogation. By providing the optical stability necessary to track the same neuronal ensembles across acquisition and expression of habits, this platform enables investigations into the computational neural dynamics underlying goal-directed and habitual behaviors.

## Introduction

Behavior is fundamentally governed by a dynamic interplay between two distinct modes of control: goal-directed actions and habits. Goal-directed actions are initially guided by action-outcome (A-O) associations, in which the value of an expected outcome drives behavior (Adams and Dickinson, 1981; Balleine and Dickinson, 1998; Dickinson and Balleine, 1999; Peak et al., 2019; Vandaele et al., 2019; Yin and Knowlton, 2006; Yin et al., 2005a; Yin et al., 2005b). This system allows for flexible behavior that can be readily adapted when circumstances change. With repeated training and reinforcement, however, this cognitively taxing approach shifts to less demanding, more automatic habitual behaviors. Here, behavior is automatically triggered by environmental cues, regardless of the outcome’s current value (Balleine and Dickinson, 1998; Yin and Knowlton, 2006; Yin et al., 2005a; Adams, 1982; Belin et al., 2009; Graybiel, 2008; Tricomi et al., 2009; Yin et al., 2004). The transition between these behavioral states is essential for efficient and adaptive behavior; however, when this balance is disrupted, and the automatic habit system becomes dominant over the flexible goal system, it can lead to maladaptive behaviors (Belin et al., 2013; Everitt and Robbins, 2016; Gillan et al., 2016; Hogarth et al., 2013; Jonkman et al., 2012; Murray et al., 2014; Vandaele and Janak, 2018; Vanderschuren et al., 2005). Understanding the neural mechanisms that govern this transition is crucial, as an imbalance favoring habitual responses is a core pathological feature across many neuropsychiatric disorders, including substance use disorder (SUD). A major challenge in this field lies in elucidating how the brain transitions from flexible, goal-directed actions to rigid habits. This lack of clarity is a major obstacle to developing effective treatments for conditions characterized by persistent, compulsive actions.

Historically, freely moving operant conditioning models have been instrumental in studying habitual behaviors (Yin et al., 2005a; Yin et al., 2004; Malvaez et al., 2018; Martiros et al., 2018; O’Hare et al., 2017; Thorn et al., 2010; Yin and Knowlton, 2004; Yin et al., 2006). However, these paradigms have limitations for the kind of longitudinal, high-resolution spatiotemporal investigation of neural circuits needed to fully capture the behavioral transition over time. To overcome this issue, researchers are increasingly combining operant conditioning paradigms with head-fixed setups and advanced neural recording technologies, such as two-photon (2P) microscopy (Clarke et al., 2024; Paniccia et al., 2024; Vollmer et al., 2021; Vollmer et al., 2022; Ward et al., 2025). While head-fixed models provide advantages for detailed, long-term neural monitoring, an experimentally validated head-fixed paradigm for operant habitual reward-seeking has yet to be developed. Establishing such a model would significantly advance our ability to observe the neural dynamics underlying goal-directed actions, habits, and the transition between the two across time.

Here, we present a novel head-fixed operant paradigm in mice that reliably produces goal-directed behavioral control with limited training and habits with overtraining. In this context, we specifically operationalize habitual control as outcome-insensitive instrumental responding, defined by the persistence of behavior despite changes in the value or the contingency of the reinforcer. By adapting well-validated tasks from freely moving models of goal-directed and habitual behavioral control (Malvaez et al., 2018), we demonstrate that mice exhibit either goal-directed or habitual responding, depending on their training duration [limited vs. extended variable-interval 30-s (VI30s) schedules]. To assess the behavioral strategy, mice were tested using the two gold-standard tests for goal-directed behavioral control, outcome devaluation and omission tasks (Balleine, 2019; Dickinson and Balleine, 1993; Malvaez and Wassum, 2018). To validate that our head-fixed paradigm engages the canonical neural substrates of habit as found in freely moving mice, we chemogenetically inhibited the dorsolateral striatum (DLS)—a region well-established as critical for habitual behavior in both rodent and human studies (Yin and Knowlton, 2006; Tricomi et al., 2009; Yin et al., 2004; Thorn et al., 2010; Yin et al., 2006; Malvaez and Wassum, 2018; Alloway et al., 2017; Graybiel and Grafton, 2015; Lipton et al., 2019; Malvaez, 2020; O’Hare et al., 2016). Together, these findings establish our head-fixed operant model as a robust and translationally relevant platform for studying goal-directed actions and habits. This model can be easily coupled with high-resolution *in vivo* imaging strategies and will provide unprecedented access to the spatiotemporal dynamics underlying the transition to, and persistence of, habitual behaviors.

## Materials and methods

### Animals

All experiments were approved by the Institutional Animal Care and Use Committee (IACUC) at the Medical University of South Carolina in accordance with the NIH-adopted Guide for the Care and Use of Laboratory Animals (Protocol 2021-01325-1). Adult (8–24 weeks) male and female C57BL/6J wild-type (WT) background mice (8 weeks old/20 g minimum at study onset; Jackson Labs or MUSC DLAR Breeding Colony) were group-housed pre-operatively and single-housed post-operatively under a reversed 12:12-h light cycle (lights off at 8:00 a.m.; all experiments were performed during the dark phase) with access to standard rodent chow and water *ad libitum*. To facilitate instrumental learning and maintain robust responding for a caloric reward (10% Ensure), mice were placed on a regulated water restriction schedule 3 days prior to the start of acquisition. This is a standard requirement for operant paradigms using liquid reinforcers to ensure that reward-seeking behavior is not prematurely extinguished by satiation (Goltstein et al., 2018; Grant et al., 2021; Guo et al., 2014; Otis et al., 2017; Otis et al., 2019). We employed a tiered supplemental water system to maintain each animal between 85 and 90% of its initial free-feeding body weight. Unlike fixed-volume restriction paradigms, our approach adjusted daily water access based on a combination of the animal’s current weight and its instrumental performance (lever presses) during the session. This method provides a more refined balance between behavioral motivation and animal welfare compared to traditional 24-h restriction protocols. To maintain mice at 85%–90% of their free-feeding body weight, we regulated their daily water intake based on their behavioral performance: 50–100 active presses: 0 mL water, 30–50 active presses: 0.25 mL water, 20–30 active presses: 0.5 mL water, 0–20 active presses: 1.0 mL water. Water was provided at least 2.5 h after the final behavioral session of the day. Mice were weighed daily before each session. If a mouse’s weight exceeded the target, its water allowance was reduced by one category to encourage weight loss. Conversely, if a mouse’s weight fell below the target, a separate set of criteria was applied to ensure adequate hydration: 50–100 active presses: 0.5 mL water, 30–50 active presses: 1.0 mL water, 20–30 active presses: 1.5 mL water, 0–20 active presses: 2.0 mL water. Mice were transitioned to a Teklad Diet (2,918, Inotiv) 1 week prior to training. This fixed-formula diet was maintained throughout the study to minimize batch-to-batch nutrient fluctuations and metabolic variability. This ensured that fluctuations in body weight were a result of the water restriction protocol rather than dietary inconsistency, providing a reliable metric for adjusting water intake.

### Surgeries

For intracranial surgeries, all mice were anesthetized with isoflurane (0.8%–1.5% in oxygen; 1 L/min) and placed within a stereotaxic frame (Kopf Instruments). Ophthalmic ointment (Akron), topical anesthetic (2% Lidocaine; Akron), analgesic (Carprofen & Ketorolac, 2 mg/kg, i.p.), and subcutaneous sterile saline (0.9% NaCl in water) were given pre- and intra-operatively for health and pain management. An antibiotic (Cefazolin, 200 mg/kg, s.c.) was given post-operatively to reduce the possibility of infection, and mice were allowed to recover for at least 2–3 weeks prior to behavioral training. All mice received head-ring implantation. A custom-made head-ring (stainless steel; 5 mm ID, 11 mm OD) was implanted onto the animal skull, along with 2–3 screws and dental cement to allow for head fixation.

#### Behavioral chemogenetics surgeries

Chemogenetic manipulations were conducted to selectively inhibit DLS neurons. During surgery, bilateral injections of a virus encoding an inhibitory DREADD (AAV2-hSyn-hM4Di-mCherry) or control (AAV2-hSyn-mCherry) were administered into the DLS (AP: +1.20, ML: +/− 1.95, DV: −3.65; 500 nL). A stainless-steel head-ring was cemented onto the skull using dental cement and skull screws. Viral placements were confirmed post-mortem via histology.

### Head-fixed operant conditioning

#### Apparatus

**Figure 1:**
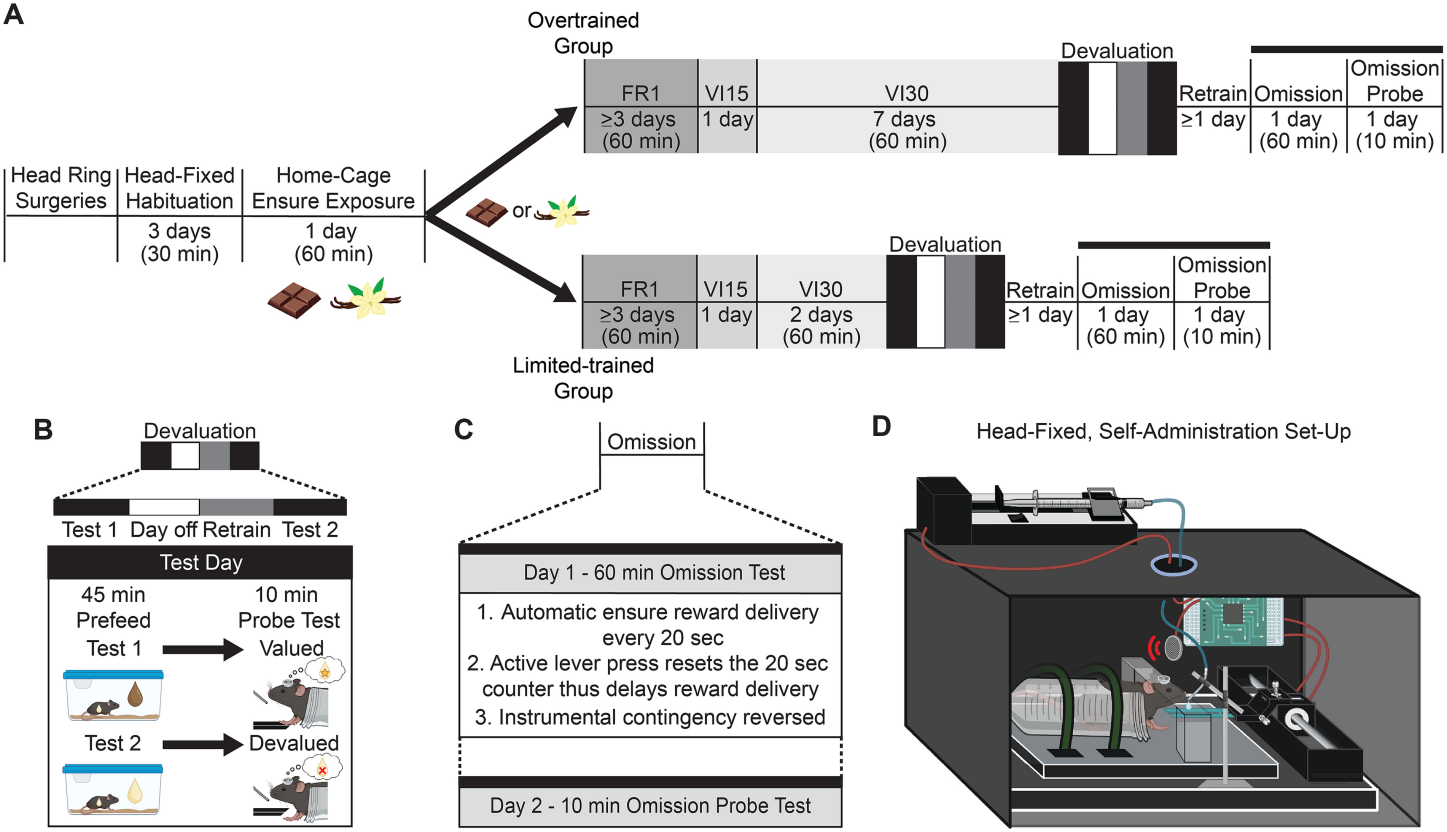
Timeline and head-fixed setup for goal-directed and habitual behavior paradigm. **(A)** Experimental timeline of head-fixed operant behavior paradigm for habitual and goal-directed behaviors. After habituation and preexposure, mice were assigned to either the overtrained or limited-trained group. Both groups received FR1, VI15s, and VI30s training; however, the overtrained group had an extended 7-day VI30s paradigm, while the limited-trained group had a short 2-day VI30s paradigm. After training, mice were tested under devaluation and omission procedures; all mice received 1 day off and VI30s retraining in between tests. **(B)** Devaluation procedure involved two test days (devalued and valued) where mice were pre-fed one flavor for 45 min in their home cage before going into the behavioral box and being tested with a 10-min probe session (no cues, no rewards). **(C)** Omission procedure consisted of two separate days. The first test day was a 60-min session where the reward was automatically delivered every 20 s. A lever press resulted in a reset of the 20-s counter. Twenty-four hours after this test, mice were tested on a 10-min probe test (no cues, no rewards) to investigate memory consolidation from the day prior. **(D)** Diagram of operant chamber and head-fixation station. Mice were fixed into the head station and placed in a restraint tube. They had access to both an active and inactive lever as well as a lick spout. Created in part with BioRender.com. Manusky, L. (2026) https://BioRender.com/4zmyix7, https://BioRender.com/wdokkzd.

Training took place in a set of 6 operant chambers that were custom-made (Figure 1D), as previously described (Vollmer et al., 2021). Behavioral systems interfaced with an Arduino and custom Python-based software (Alloway et al., 2017; Clarke et al., 2024; Paniccia et al., 2024; Vollmer et al., 2021; Vollmer et al., 2022; Ward et al., 2025) to deliver liquid reinforcers (2 s), play a tone cue (8 kHz, 1 s), record lever presses (active/inactive), and collect lick contacts. During behavioral acquisition, levers were placed in front of the mice. Pressing the active lever resulted in immediate cue presentation, followed by reward delivery (12.5 μL; 10% Ensure mixed in tap water) delivered at the spout. Pressing the inactive lever produced no cue or reward. Each instrumental training session lasted 60 min, during which mice were allowed to freely acquire Ensure for the entirety of the session.

#### Natural reward

All operant conditioning experiments involved reward delivery of 10% Chocolate or Vanilla Ensure Original (Abbott Nutrition, Columbus, OH). Mice were randomly assigned and equally counterbalanced to earn one of the Ensure flavors throughout training (i.e., training flavor). At least 1 day prior to the start of instrumental studies, mice received Ensure preexposure (60-min home-cage *ad libitum* access of chocolate and vanilla Ensure). By offering mice unrestricted access to both flavors of Ensure in their home cages for at least 1 day before instrumental studies, we aimed to diminish any behavioral responses driven by novelty, ensuring subsequent observations more accurately reflected the behavioral preferences. In addition, all mice were given equated exposure (i.e., non-contingent access to the alternative Ensure flavor) in a different room for a week prior to the start of training to diminish novelty of the alternative Ensure flavor.

A separate cohort of mice (*n* = 8) was tested to confirm discriminability between 10% chocolate and vanilla Ensure and to rule out baseline flavor preference. Mice were given 2 h of *ad libitum* access to both flavors in the home cage, and consumption did not differ (Supplementary Figures S1A,B). To assess discrimination, mice underwent two sensory-specific devaluation tests. Following a 90-min pre-feeding session with one flavor (devalued) (Supplementary Figure S1C), they received 10 min of *ad libitum* access to both flavors. Mice consumed significantly more of the valued flavor, confirming the ability to discriminate between flavors (Supplementary Figures S1D,E).

#### Acquisition

To reduce possible stress induced by head-fixation, mice underwent 3 days of habituation during which they were head-restrained in custom-built operant chambers (Vollmer et al., 2021) for 30 min with no access to the levers, tone cue, or reward. Following habituation, mice were separated into the (1) limited-trained group or the (2) overtrained group and trained on a fixed-ratio 1 (FR1) schedule of reinforcement until they pressed a total of 50 times on the active lever, 2 days in a row (60-min sessions) for delivery of their training flavor (chocolate or vanilla). Each session began with guiding the mouse into the head-fixation station and placing their body into a restraint tube, to decrease bodily movement. Both the active and inactive levers, along with a lick spout, were then positioned in front of the mouse, requiring the mouse to extend their forelimbs in order to press either lever (Vollmer et al., 2021). Mice received one training session per day, with sessions conducted on consecutive days, including weekends, to ensure continuous training without rest periods. Mice required an average of 5.2 ± 0.4 days (mean ± SEM) to meet the FR1 acquisition criteria (range: 3–12 days). After mice achieved performance criteria (50 presses/60 min, 2 days in a row), they progressed to the next stage of the paradigm.

#### Variable-interval training

After completing FR1 training, all mice were shifted to a variable-interval 15-s (VI15s) reinforcement schedule for 1 day. Mice designated for limited training were then trained on a VI30s schedule for 2 days, while mice designated for overtraining remained on VI30s for 7 days (Figure 1A). Although some paradigms use longer durations, this 7-day “overtraining” protocol aligns with established mouse models (Malvaez and Wassum, 2018; Giovanniello et al., 2025; Malvaez et al., 2025) and contemporary studies demonstrating that extended interval-based reinforcement schedules are sufficient to induce outcome-insensitivity (Gremel and Costa, 2013; Rossi and Yin, 2012). We utilized a consistent VI30s schedule for both groups to isolate training duration as the primary variable, establishing a standardized platform for future longitudinal imaging without the confounding rule shifts associated with RR-to-VI transitions. All sessions lasted 60 min. Following training, mice were tested for goal-directed or habitual behavioral strategies via devaluation and omission tests.

#### Devaluation test

To assess sensory-specific satiety using the devaluation test, mice received two test days (devalued and valued), with the order counterbalanced across groups. On each test day, mice were first given a 45-min prefeed period of unlimited access to either the flavor earned during training (devalued) or the alternative, pre-exposed flavor (valued), with the latter controlling for general satiety. Consumption during this prefeed period did not differ between control and experimental groups (Supplementary Figure S2B). Immediately following this prefeed, lever pressing was measured during a 10-min, non-reinforced probe test (Figure 1B). Between test days, mice received an off day followed by retraining on VI30s, until mice reached at least 80% of their baseline lever press rate before proceeding to the subsequent test. Baseline was determined from the last day or the average of the last two VI30s training sessions. If one of the last 2 days exceeded two standard deviations from the overall mean, baseline was instead calculated using the last 3 days. The order of test days was counterbalanced across mice. In addition to raw press rates, a devaluation index was calculated for each subject: $\text{Devaluation Index}=\frac{\left(\#\text{of devalued lever presses}\right)}{\left(\#\text{of devalued}+\text{valued lever presses}\right)}$ . This index was used to account for variability in press rates between subjects.

#### Omission test

After devaluation testing, mice were given a day off and retrained as described above. Once retraining criteria were met, mice underwent an omission test and a subsequent omission probe test (Figure 1C). The omission test was a 60-min session in which the lever-outcome contingency was reversed: Ensure was delivered non-contingently every 20 s, and any active lever press reset the timer, delaying reward delivery (Rossi and Yin, 2012). Twenty-four hours later, lever pressing was measured during a 10-min, non-reinforced omission probe test to assess the persistence of the learned behavioral change.

### Data collection and statistical analyses

Lever press and lick data during acquisition and test sessions were collected using a custom Python interface, REACHER, connected to Arduino (Vollmer et al., 2021). Statistical analyses were performed using GraphPad PRISM (Dotmatics, version 10). Behavioral data were analyzed using either analysis of variance (ANOVA; two-way or three-way) or t-tests (paired or unpaired), as appropriate. The independent variables analyzed included: lever type (active or inactive), sex (male or female), virus (mCherry vs. DREADD), training duration (limited vs. overtrained), and day (acquisition, devaluation test, omission test). Sidak’s and Tukey *post hoc* tests were used following significant main effects and interactions with the ANOVA analyses. The two-stage linear step-up procedure of Benjamini, Krieger, and Yekutieli (BKY) was also used, as appropriate, for controlling the False Discovery Rate in multiple comparisons. Prior to analysis, all datasets were checked for normality and homogeneity of variance.

**Figure 2:**
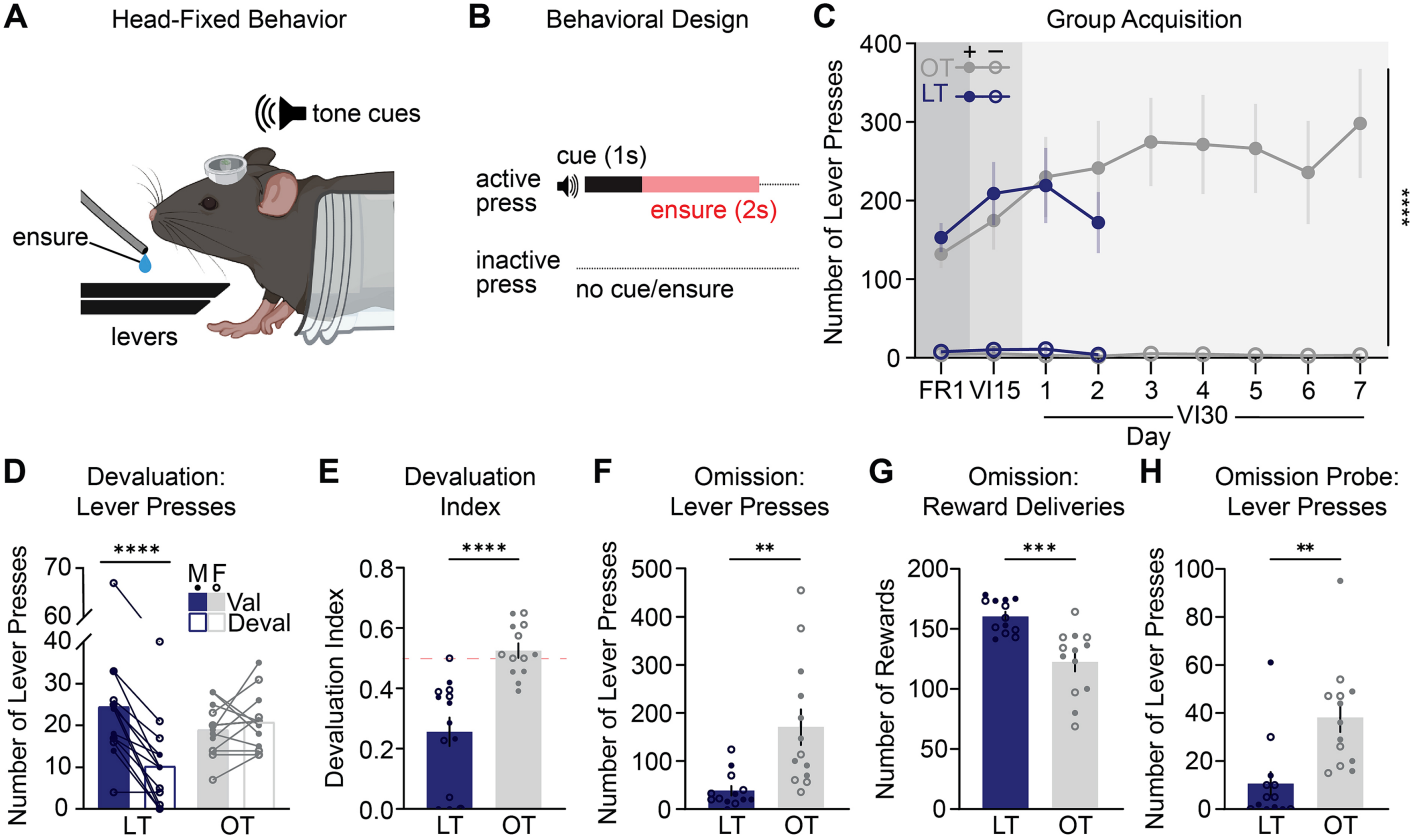
Head-fixed mice reliably acquire instrumental lever pressing and display both goal-directed and habitual behaviors. **(A)** Zoomed in schematic of head-fixed operant behavior setup. **(B)** Behavioral design, where an active lever press triggers a 1 s tone cue followed by a 2 s delivery of 10% Ensure and an inactive lever press results in no cue/Ensure. **(C)** Grouped data of active (filled circles) and inactive (open circles) lever presses across acquisition days. The FR1 data point represents the last FR1 training session for each mouse. Mice reliably discriminate between levers, pressing significantly more on the active lever (*n* = 27: 14M, 13F; 3-way ANOVA, lever effect: F_1,25_ = 58.25, *p* < 0.0001, day x lever interaction: F_1.81,45.16_ = 3.723, *p* = 0.0359; OT = overtrained, LT = limited-trained). **(D)** Devaluation tests showed LT mice pressed significantly more on the valued day than the devalued day, whereas OT mice showed no difference between days (*n* = 27: 14M = filled dots, 13F = unfilled dots; two-way ANOVA, training x test day interaction: F_1,25_ = 29.57, *p* < 0.0001, Sidak’s *post hoc* test, LT: *p* < 0.0001, OT: *p* = 0.6323). **(E)** Devaluation index significantly differed between groups; scores closer to 0.5 (red dashed line) indicate insensitivity to devaluation. LT mice were more sensitive to outcome devaluation than OT mice (*n* = 27: 14M, 13F; t(25) = 4.98, *p* < 0.0001). **(F,G)** Omission test wherein **(F)** active lever pressing was increased (*n* = 26; 13M, 13F; t(24) = 3.48, *p* = 0.0019) and **(G)** reward deliveries were reduced in the OT group (t(24) = 4.40, *p* = 0.0002). **(H)** Omission probe test showed a significant increase in lever pressing in the OT group (*n* = 26; 13M, 13F; t(24) = 3.55, *p* = 0.0016). See Supplementary Table S1 for complete analyses. All data are presented as mean ± SEM. **p < 0.01, ***p < 0.001, ****p < 0.0001. Created in BioRender. Manusky, L. (2026) https://BioRender.com/3xqnb2m.

To ensure findings were robust against between-subject variability and missing values, key longitudinal datasets (e.g., acquisition) were cross-validated using linear mixed-effects models (REML), which yielded results consistent with our reported ANOVA findings. For analyses in Figure 2, mCherry control mice from chemogenetic cohorts were pooled with the original acquisition cohorts, as behavioral protocols were identical. Sample sizes were determined based on established standards in the field for chemogenetic behavioral studies. The adequacy of these sample sizes is supported by the robust effect sizes (partial eta squared) observed across all primary behavioral interactions. Mouse exclusion criteria were defined *a priori*. Specifically, (1) failing FR1 acquisition within 14 days; (2) loss of head-cap stability (3); retraining exceeding 7 days; or (4) viral expression outside the DLS. Altogether, one mouse was excluded during FR1 training, and another was excluded from omission testing, both due to head-cap instability.

### Behavioral chemogenetics

To investigate the role of the DLS in regulating goal-directed and habitual reward-seeking behaviors, we used a site-specific chemogenetic approach. Mice were first trained on the limited 2-day or extended 7-day head-fixed instrumental paradigm, as described above. Upon successful completion of this training, mice received a systemic pretreatment of clozapine *N*-oxide [CNO, 10 mg/kg; i.p.; (Mahler et al., 2019)] 30 min before each test session, which included devaluation, valuation, omission, and omission probe. During devaluation test days, CNO was administered 15 min after the onset of the pre-feeding period. This timing was selected to ensure that peak DREADD-mediated inhibition coincided with the instrumental devaluation test. Importantly, DLS inhibition did not affect the pre-feeding process itself; no differences in the amount of Ensure consumed were observed between DREADD-expressing mice and controls [t(76) = 1.579, *p* = 0.1184], nor between the experimental groups and prior pilot cohorts [t(54) = 0.006, *p* = 0.9949].

After behavioral testing, mice were transcardially perfused, and brains were collected and post-fixed in 4% paraformaldehyde (PFA). For cryoprotection, brains were subsequently immersed in successive 15 and 30% sucrose solutions. Coronal sections (40 μm) were cut using a cryostat, mounted on slides, and imaged on a Leica THUNDER Imager Tissue.

## Results

### Acquisition of head-fixed goal-directed and habitual reward-seeking

Previous freely moving instrumental models have successfully produced both goal-directed and habitual behavioral control (Adams and Dickinson, 1981; Yin et al., 2006; Gremel and Costa, 2013; Corbit et al., 2012; Jones et al., 2022). However, a head-fixed model capable of capturing each behavioral strategy in isolation has not yet been developed. To this end, here head-fixed mice were trained to press an active lever to receive a 1 s tone cue paired with delivery of 10% (v/v) Ensure (Figures 2A,B). A second inactive lever was present but did not result in a cue or reward. To establish the desired goal-directed or habitual strategies using a paradigm adapted from well-validated models (Malvaez et al., 2018), training progressed across three different schedules of reinforcement: FR1, VI15s, and VI30s. VI schedules were included to promote habitual control, as the unpredictability of reinforcement reduces reliance on immediate A-O associations and promotes the development of stable, outcome-insensitive behavior. To assess initial learning trajectory before the divergence in training protocols, we compared the performance of limited and overtrained groups across their shared acquisition phase. Mice in both groups pressed the active lever significantly more than the inactive, revealing comparable initial lever discrimination and overall lever press rates that reflected the progressive development of robust discrimination between the active and inactive levers (Figure 2C). This acquisition pattern was further validated by a linear mixed-effects model, which confirmed the primary effects of Lever (*p* < 0.0001) and Day (*p* = 0.028) while accounting for individual subject variability. Assessment of sex differences revealed no significant main effect of sex on active lever press rates (Supplementary Figure S2A). Overall, these data confirm successful and comparable initial acquisition of instrumental behavior across all animals prior to the manipulation of training duration.

### Devaluation tests differentiate goal-directed and habitual behavior

After completing acquisition on their respective schedules, mice received a set of devaluation tests to assess their underlying behavioral strategy. In these tests, non-reinforced lever pressing was measured after a 45-min prefeed period with the Ensure flavor earned during training (devalued condition) and compared to pressing after pre-feeding with the alternative Ensure flavor provided outside the training context (valued condition). The order of the devalued and valued conditions was counterbalanced across subjects and each mouse experienced both conditions, allowing direct within-subject comparison of responding during the valued versus devalued state. Goal-directed actions are based on knowledge of the A-O relationship and are therefore flexible, resulting in a reduction of responding when the outcome is devalued. In contrast, habits are marked by a lack of consideration of action consequences, leading to inflexibility, and persistent responding regardless of outcome’s current value. To ensure devaluation wasn’t simply due to varying Ensure intake during prefeed, we measured prefeed consumption and found no difference across conditions or groups (Supplementary Figure S2B).

During devaluation tests, limited-trained mice demonstrated sensitivity to the changing value of the outcome, exhibiting a significantly lower lever-press rate on the devalued test day compared to the valued test day. In contrast, overtrained mice showed no change in lever press rate across the valued and devalued test days, demonstrating insensitivity to outcome devaluation (Figure 2D). To further quantify this behavioral distinction, a devaluation index was calculated (# of devalued lever presses) / (# of devalued + valued lever presses). This index provides a normalized measure of goal-directed versus habitual control, where 0.5 indicates insensitivity to devaluation (habitual control) and 0.0 indicates sensitivity to devaluation (goal-directed control). Consistent with the overall press rates, the devaluation index in limited-trained mice was reduced as compared with overtrained mice, confirming that limited-trained mice were selectively sensitive to reward devaluation (Figure 2E). The number of licks for the valued and devalued test days did not differ (Supplementary Figure S2C), suggesting consumption did not change and that the observed differences were specific to the instrumental response.

### Omission tests reveal group differences in contingency sensitivity

Following devaluation testing and subsequent retraining, mice received an omission test designed to test their behavioral flexibility by changing the A-O relationship. In this procedure, rewards were delivered automatically every 20 s, any lever press reset the timer, thereby delaying reward delivery. This test differentiates goal-directed actions from habits: limited-trained mice, relying on their knowledge of the A-O contingency, should rapidly reduce pressing to maximize the automatic rewards. In contrast, overtrained mice—whose actions are independent of the A-O contingency—are expected to maintain high press rates, demonstrating inflexibility. During the 60-min test, limited-trained mice showed behavioral flexibility by significantly reducing lever pressing in response to the reversed contingency. In contrast, overtrained mice were inflexible, continuing to press at high rates and failing to adjust their behavior despite the absence of reward (Figure 2F). Consistent with their persistent responding, overtrained mice received significantly fewer reward deliveries than limited-trained mice (Figure 2G). The number of licks for the omission test did not differ between the groups (Supplementary Figure S2D), suggesting consumption was not a contributing factor to the difference in lever pressing.

Twenty-four hours after the initial omission test, all mice completed a 10-min, non-reinforced omission probe test to assess memory consolidation of the new, reversed A-O contingency. Mice with limited training maintained low lever pressing rates, consistent with retention of the altered A-O contingency from the previous day. Conversely, overtrained mice continued to press at high rates, indicating persistent insensitivity to the prior omission contingency and a failure to adapt their behavior despite prior experience (Figure 2H). The number of licks during this non-reinforced omission probe test did not differ between the groups (Supplementary Figure S2E). In summary, these results demonstrate that our novel head-fixed instrumental learning paradigm successfully recapitulates the two distinct modes of behavioral control seen in freely moving models. The use of both devaluation and omission tests confirmed a clear divergence in behavioral strategy, with the limited-trained group exhibiting flexible, goal-directed actions and the overtrained group displaying inflexible habits.

### DLS neuronal activity is necessary for the expression of habitual behavior

**Figure 3:**
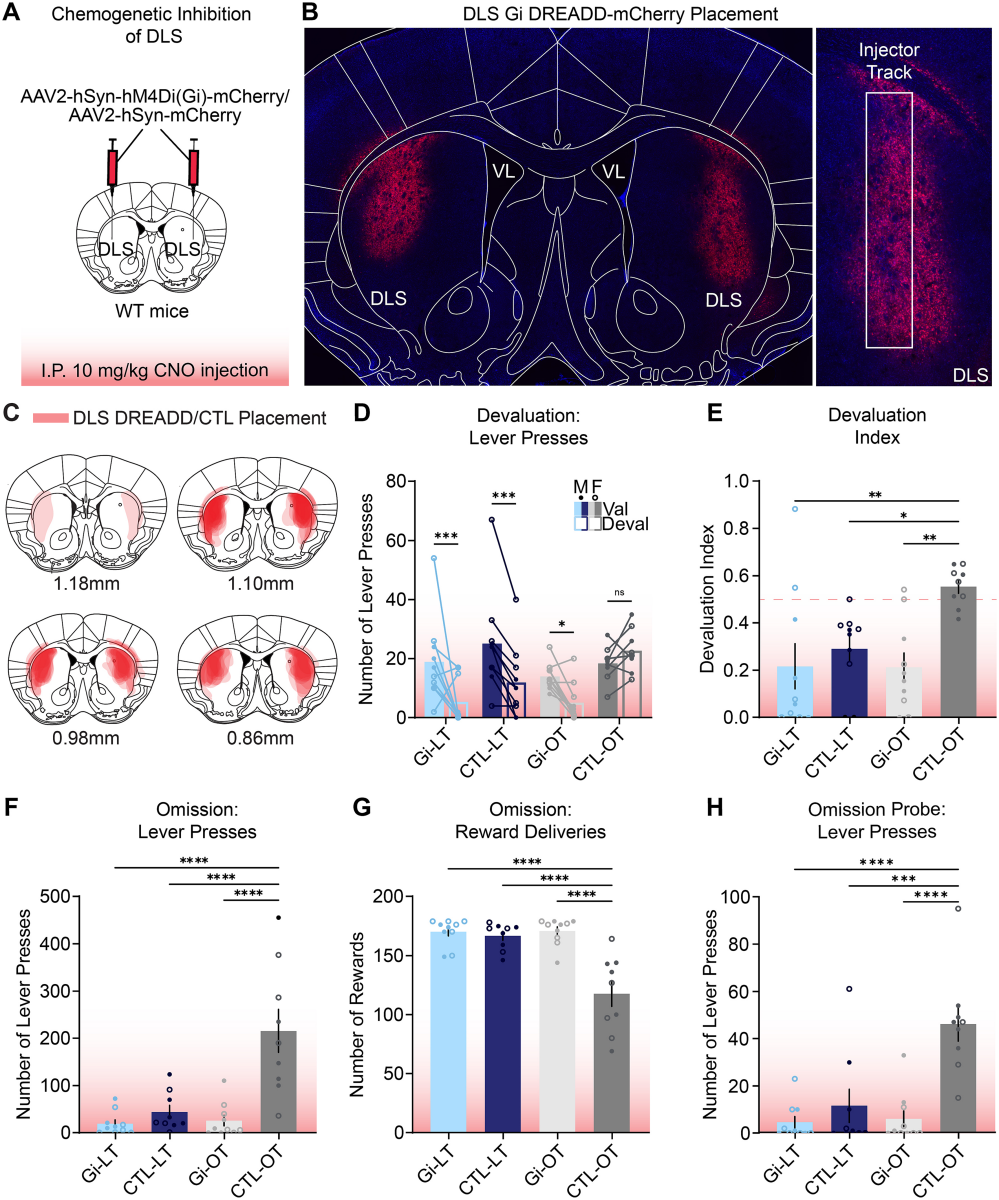
Chemogenetic inhibition of DLS suppresses habitual responding and restores goal-directed responding. **(A)** Schematic of surgical strategy enabling bilateral Gi-DREADD expression in DLS neurons. Experimental groups included control (CTL; mCherry) and inhibitory DREADD (Gi; hM4Di-mCherry) mice with either limited training (LT) or overtraining (OT). All mice received 10 mg/kg CNO via intraperitoneal (i.p.) injection 30 min before each test session. **(B)** Representative image of viral placement in DLS (left), including zoomed image of injector tract (right, DAPI = blue; mCherry = red). **(C)** Placement map of all DREADD and control viruses. **(D)** Devaluation tests showed CTL-OT mice exhibited no difference between valued and devalued days, confirming insensitivity to devaluation. In contrast, CTL-LT, Gi-LT, and Gi-OT mice all had significantly greater responding on the valued versus devalued day (*n* = 39: 19M = filled dots, 20F = unfilled dots; three-way ANOVA, test day x training interaction: F_1,35_ = 9.756, *p* = 0.0036, two-stage BKY post hoc test, Gi-LT: *p* = 0.0004, CTL-LT: *p* = 0.0006, CTL-OT: *p* = 0.2550, Gi-OT: *p* = 0.0145). The CTL-OT mice pressed significantly more than the Gi-OT mice on the devalued day (*p* = 0.0004), statistical result not shown. **(E)** Devaluation index significantly differed between CTL-OT mice and all other groups (*n* = 39: 19M, 20F; two-way ANOVA, interaction effect: F_1,35_ = 4.196, *p* = 0.0481, Tukey *post hoc* tests, Gi-LT: *p* = 0.0052, CTL-LT: *p* = 0.0378, Gi-OT: *p* = 0.0047). **(F)** Omission test showed Gi-LT, CTL-LT, and Gi-OT mice significantly reduced active lever pressing compared to CTL-OT mice. (*n* = 38: 19M, 19F; two-way ANOVA, interaction effect: F_1,34_ = 12.16, *p* = 0.0014, Tukey *post hoc* tests, Gi-LT: *p* < 0.0001, CTL-LT: *p* < 0.0001, Gi-OT: *p* < 0.0001). **(G)** Similarly, for the number of reward deliveries received, the CTL-OT group received significantly fewer rewards than the Gi-OT, Gi-LT, and CTL-LT groups, consistent with their persistent, unrewarded pressing (*n* = 38: 19M, 19F; two-way ANOVA, interaction effect: F_1,34_ = 16.71, *p* = 0.0003, Tukey post hoc tests, Gi-LT: *p* < 0.0001, CTL-LT: *p* < 0.0001, Gi-OT: *p* < 0.0001). **(H)** Omission probe test wherein Gi-LT, CTL-LT, and Gi-OT mice significantly reduced active lever pressing relative to CTL-OT mice (*n* = 38: 19M, 19F; two-way ANOVA, interaction effect: F_1,34_ = 9.961, *p* = 0.0033, Tukey *post hoc* tests, Gi-LT: *p* < 0.0001, CTL-LT: *p* = 0.0004, Gi-OT: *p* < 0.0001). See Supplementary Table S1 for complete analyses. All data are presented as mean ± SEM. *p < 0.05, **p < 0.01, ***p < 0.001, ****p < 0.0001.

To further verify the utility of this head-fixed approach, we assessed whether the habitual behavior would rely on the same canonical neuronal systems that have been described in freely moving studies. Thus, using chemogenetics we inhibited neuronal activity in the DLS during devaluation and omission tests in both limited-trained and overtrained mice. WT mice received bilateral DLS microinjections of either a Gi-coupled (Gi) DREADD (AAV2-hSyn-hM4Di-mCherry) or a control (CTL) virus (AAV2-hSyn-mCherry) (Figures 3A–C). Prior to each testing session (30 min), systemic administration of CNO (10 mg/kg, i.p.) was performed to activate the DREADD. Prefeed consumption was again measured and did not differ across groups or test day (Supplementary Figure S3A).

During devaluation testing, Gi-limited, CTL-limited, and notably, Gi-overtrained mice all demonstrated sensitivity to devaluation by significantly reducing lever presses on the devalued day compared to the valued day. Conversely, CTL-overtrained mice showed no reduction (Figure 3D). An effect of the DLS neuronal inhibition was further seen in Gi-overtrained mice pressing significantly less than CTL-overtrained mice on the devalued day, indicating that chemogenetic inhibition of the DLS prevented habit expression and restored goal-directed control in overtrained animals (Figure 3D, statistical result not shown). To account for individual differences in lever press rates, a devaluation index was calculated. CTL-overtrained mice displayed a higher index—indicating insensitivity to devaluation—compared to Gi-overtrained, Gi-limited, and CTL-limited mice (Figure 3E). Thus, Gi-overtrained mice exhibited sensitivity to devaluation, confirming that chemogenetic inhibition of the DLS restored goal-directed control in overtrained mice. Analysis of lick data showed that only the Gi-limited group displayed a significant difference in the number of licks between the valued and devalued test days, while all other groups showed no difference (Supplementary Figure S3B).

Next, we examined how chemogenetic DLS inhibition affected responding in omission and omission probe tests. In the omission test, we found that Gi-limited, CTL-limited, and notably, Gi-overtrained mice significantly reduced lever pressing and received more reward deliveries than the CTL-overtrained mice (Figures 3F,G). This pattern of responding demonstrates that Gi-overtrained mice, but not CTL-overtrained controls, regained sensitivity to changes in the instrumental contingency following chemogenetic inhibition of the DLS. This distinction persisted into the omission probe test a day later, where Gi-limited, CTL-limited, and Gi-overtrained mice maintained low press rates, while CTL-overtrained mice continued pressing at high rates despite the absence of the cue tone or reward (Figure 3H). In both omission and omission probe tests, lick data showed no change between any of the groups (Supplementary Figures S3C,D), confirming that the observed differences in pressing rates were due to behavioral adaptations and not consumption differences. These results demonstrate that DLS activity is required for the expression of outcome-insensitive responding in overtrained mice, as silencing this region reinstated goal-directed flexibility during both devaluation and omission tests.

Collectively, these results demonstrate that while CTL-overtrained mice continued to exhibit inflexible, habitual responding, the Gi-limited, CTL-limited, and, critically, the DLS-inhibited Gi-overtrained mice all displayed behavioral flexibility, significantly reducing their lever pressing when it no longer resulted in a valued reward. These findings provide strong evidence that chemogenetic inhibition of the DLS suppresses habitual behavior and restores goal-directed control, further validating our paradigm for modeling goal-directed and habitual actions.

## Discussion

This study introduces and validates a novel head-fixed operant paradigm in mice that reliably produces flexible, goal-directed behavioral control with limited training and inflexible habitual behavior with overtraining. Mice in this paradigm acquired instrumental responses consistently and effectively discriminated between active and inactive levers across different reinforcement schedules. The training regimen determined the behavioral strategy: overtrained mice continued lever pressing despite outcome devaluation and did not reduce pressing when rewards were omitted, whereas mice with limited training adapted their behavior when outcome value or A-O contingencies changed. Importantly, chemogenetic inhibition of the DLS in overtrained mice prevented the expression of habits, restoring sensitivity to devaluation and contingency reversal. This demonstrates that DLS activity is essential for maintaining habits and that these mice regained the ability to flexibly adjust behavior based on changes in expected outcome. These results validate our head-fixed operant paradigm as an effective model for studying both goal-directed and habitual behavior and demonstrate its utility for integration with advanced technologies that require head-fixation, such as *in vivo* imaging and precise circuit manipulations.

### Advancing habit research with head-fixed models

Traditionally, studies of habitual behavior have relied on freely moving operant conditioning, which, while valuable, limits the spatiotemporal resolution and longitudinal tracking of individual neurons necessary for fully capturing the habit formation process. Recently, head-fixed setups have been adapted for various drug and reward self-administration tasks, allowing detailed neural monitoring during goal-directed learning (Clarke et al., 2024; Paniccia et al., 2024; Vollmer et al., 2021; Vollmer et al., 2022; Ward et al., 2025). Our work builds on this progress by showing that mice in a head-fixed setup not only learn goal-directed actions but can also transition to and sustain habitual behavior. Behavioral outcomes in our paradigm recapitulate key features of habitual control observed in freely moving animals, including insensitivity to outcome devaluation and persistent responding under altered contingencies. Crucially, this paradigm provides a methodological framework for addressing scientific questions that have remained inaccessible in freely moving models. The mechanical stability afforded by head-fixation allows for the longitudinal tracking of neural ensembles across the multi-day transition from goal-directed to habitual states. This level of access facilitates cell-level interrogation of the specific circuit dynamics and population shifts that occur as behaviors consolidate into outcome-insensitive habits. This establishes our system as a powerful, analogous platform for dissecting the neural circuit adaptations underlying habits.

### Benefits of 2P microscopy for neural dynamics of habitual behavior

Our head-fixed paradigm’s compatibility with high-resolution, 2P microscopy provides an opportunity to observe the precise neural adaptations that drive the transition to habitual behavior. 2P microscopy offers superior resolution, deeper tissue penetration, and reduced phototoxicity, enabling prolonged *in vivo* imaging sessions. This setup provides stable, longitudinal optical access to specific neural populations, allowing us to track cellular and synaptic changes as behaviors consolidate into habits. Crucially, this combined approach enables the direct correlation of single-neuron activity and population dynamics with the moment-to-moment emergence and expression of goal-directed actions and habits. Such detailed, time-resolved tracking of circuit activity during habit formation—including the mapping of dynamic neural codes across learning stages and the identification of specific cells recruited during the transition—has been largely inaccessible with previous methodologies. This level of interrogation will be essential for fully elucidating the underlying circuit mechanisms of behavioral inflexibility and rigidity.

### Causal role of the DLS in habit persistence

Our functional manipulations provide strong support for the established role of the DLS in habitual behavior and, crucially, validate the fidelity of our head-fixed paradigm. The DLS is widely recognized as a critical hub for habit expression. We found that chemogenetic inhibition of the DLS in overtrained mice disrupted the expression of inflexible habits, restoring sensitivity to outcome devaluation and contingency reversal. While these results—that DLS activity is necessary for maintaining habits and that its modulation can reinstate goal-directed, flexible control—are consistent with findings from freely moving models (Balleine, 2019; Dickinson and Balleine, 1993; Malvaez and Wassum, 2018), our study extends this work by demonstrating that DLS activity is acutely necessary for the real-time expression of habits. Unlike foundational studies that utilized permanent lesions—thereby affecting both the acquisition and maintenance of habits—our results show that DLS activity is required to maintain the outcome-insensitive state even after a habit has been fully established. By showing that DLS modulation can reinstate behavioral flexibility, our work highlights the DLS as a potent therapeutic target for restoring goal-directed control.

### Methodological considerations of reinforcement logic

A unique feature of this paradigm is the use of a consistent VI30s schedule for both limited-trained and overtrained cohorts, rather than the canonical random ratio (RR) to VI dissociation often employed in freely moving studies (Yin et al., 2005a; Malvaez et al., 2018). This VI → VI design was a deliberate methodological choice to isolate training duration as the sole independent variable. By maintaining a single reinforcement logic, we establish a standardized platform for future longitudinal imaging that avoids the confounding neural and behavioral shifts associated with rule transitions. This approach allows for the direct comparison of early-stage versus late-stage VI performance, facilitating the identification of circuit-level shifts that occur as behaviors consolidate into habits. While VI schedules are traditionally associated with accelerated habit formation, our limited-trained mice remained robustly sensitive to both sensory-specific devaluation and contingency omission. This empirical validation confirms that the limited-trained group maintains an A-O associative structure, making them a true goal-directed comparison for the overtrained habit phenotype. Furthermore, modeling the transition within a single VI schedule may more closely recapitulate the graded erosion of behavioral flexibility observed in naturalistic and pathological states, where habits often emerge from consistent, time-based reinforcement rather than shifts in work-to-reward ratios.

### Limitations and future directions

While the head-fixed instrumental learning paradigm presented here offers opportunities for investigating the computational neural dynamics of habitual behavior, it is important to acknowledge certain inherent limitations common to such *in vivo* models. While head restraint can potentially induce stress, the successful acquisition of flexible, goal-directed actions, comparable to freely moving studies, strongly suggests adequate acclimation and low confounding stress levels (Giovanniello et al., 2025). Future integration of air-lifted platforms could further enhance validity and physiological monitoring. Additionally, while we utilized a consistent VI schedule to isolate the temporal transition from flexible action to habit, we acknowledge that RR schedules classically sustain goal-directed behavior for longer durations. Comparing the neural signatures of our goal-directed VI cohorts to RR-trained animals remains an important future direction to determine how reinforcement logic interacts with the rate of habit consolidation. The simplified motor requirements of head-fixation may also reduce motivational demands relative to freely moving models; future work could introduce high response requirements or more complex task structures to better engage motivated behavior. Furthermore, because the motor requirements of head-fixation are simplified and constrained, this model does not directly assess sensorimotor dimensions of habit related to motor topography, such as the development of automated motor sequences or action chunking. Future iterations of this head-fixed preparation will allow for the integration of high-resolution tracking to investigate how these complex motor features emerge alongside outcome-insensitivity. Despite these considerations, this paradigm provides a robust framework for examining the transition to, and maintenance of, habitual behavior with unparalleled temporal and spatial resolution. Collectively, these identified limitations offer valuable avenues for future model refinement and expansion.

## Conclusion

Our results introduce and validate a novel head-fixed operant paradigm that reliably models both goal-directed and habitual behavior in mice. This paradigm demonstrates the transition to and maintenance of habitual control and shows that DLS activity is critically required for sustaining the expression of habits. Importantly, inhibition of the DLS restores behavioral flexibility, allowing animals to adjust actions based on outcome value and contingency changes. Our model provides access to high-resolution neural dynamics, paving the way for deeper insights into habit formation and maintenance, and informing targeted therapeutic strategies for maladaptive habitual actions.

## Data Availability

The raw data supporting the conclusions of this article will be made available by the authors, without undue reservation.

## Supplementary material

SUPPLEMENTARY FIGURE S1 Initial 10% chocolate and vanilla Ensure preference and discrimination. **(A)** Consumption of vanilla (light yellow bar) and chocolate (dark brown bar) during the initial preference test. No difference in consumption for each flavor [*t*(7)=0.35, *p* = 0.7398]. **(B)** Percentage preference for vanilla and chocolate, calculated based on the consumption shown in **(A)**. No difference in preference for each flavor [*t*(7)=0.38, *p* = 0.7122]. **(C)** Consumption of vanilla and chocolate during the 90-minute prefeed phase, used to selectively devalue one of the flavors. Over the course of both tests there was no difference in prefeed consumption [*t*(7)=0.27, *p* = 0.7939]. (**D**) Consumption of the valued flavor and the devalued during a 10-minute probe test following the prefeed. Consumption of the devalued flavor was significantly lower than the valued flavor [*t*(15)=4.48, *p* = 0.0004], indicating successful devaluation. **(E)** The calculated devaluation index for each subject. Devaluation index (devalued consumption) / (devalued consumption + valued consumption), scores closer to 0.5 (red dashed line) indicate insensitivity to devaluation and scores closer to 0.0 indicated sensitivity to devaluation. See Supplementary Table S1 for complete analyses. All data are presented as mean ± SEM.

SUPPLEMENTARY FIGURE S2 Sex comparison, prefeed consumption, and lick data across tests. **(A)** Number of lever presses over training paradigms, comparing males (blue) and females (pink), as well as active (filled dot) and inactive (unfilled dot) lever presses. Lever pressing significantly increased across days, with a robust difference observed between the active and inactive lever. Males and females exhibited no significant difference in overall press rate (*n* = 27: 14M, 13F; mixed effects, three-way ANOVA, lever effect: F_1,25_ = 153.9, *p* < 0.0001, sex effect: F_1,60_ = 1.974, *p =* 0.1652, day x lever interaction: F_1.26,9.44_ = 5.274, *p* = 0.0401). **(B)** Consumption of the valued (filled bar) and the devalued (unfilled bar) flavor during the pre-feeding period did not differ between groups. **(C)** Number of licks during the valued and devalued test days did not differ between groups. **(D)** Total number of licks during the omission test in the limited-trained and overtrained groups did not differ. **(E)** Total number of licks during brief 10-minute omission probe test did not differ between groups. See Supplementary Table S1 for complete analyses. All data are presented as mean ± SEM.

SUPPLEMENTARY FIGURE S3 DREADD prefeed and lick data. **(A)** Consumption of the valued (filled bar) and devalued (unfilled bar) flavor during the prefeed period, across all groups. Limited-trained animals and overtrained animals show similar consumption patterns. **(B)** The total number of licks for the valued and devalued test, Gi-LT mice licked significantly less on the devalued day compared to the valued day, with no difference found for any of the other groups (*n* = 39: 19M, 20F; three-way ANOVA, test day effect: F_1,35_ = 9.110, *p* = 0.0047, Two-stage BKY *post hoc* test, Gi-LT: *p* = 0.0342). **(C)** Total number of licks during the omission test, no difference between groups. **(D)** Total number of licks during omission probe test, no difference between groups. See Supplementary Table S1 for complete analyses. All data are presented as mean ± SEM.

SUPPLEMENTARY TABLE S1 Supplementary statistical table.
