# Supplementary figures and images for: Goal-directed actions and habits in head-fixed mice
Source: Front Behav Neurosci. 2026 Feb 25;20:1751553. doi: 10.3389/fnbeh.2026.1751553 (PMC12975963; doi:10.3389/fnbeh.2026.1751553)

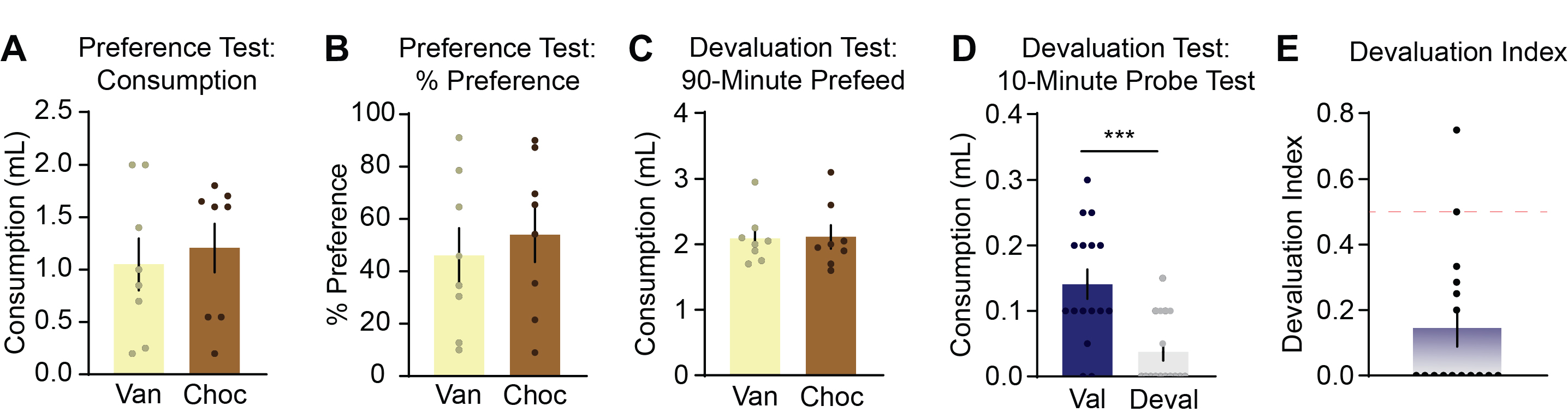

Supplement: SUPPLEMENTARY FIGURE S1 — Initial 10% chocolate and vanilla Ensure preference and discrimination. (A) Consumption of vanilla (light yellow bar) and chocolate (dark brown bar) during the initial preference test. No difference in consumption for each flavor [t(7)=0.35, p = 0.7398]. (B) Percentage preference for vanilla and chocolate, calculated based on the consumption shown in (A). No difference in preference for each flavor [t(7)=0.38, p = 0.7122]. (C) Consumption of vanilla and chocolate during the 90-minute prefeed phase, used to selectively devalue one of the flavors. Over the course of both tests there was no difference in prefeed consumption [t(7)=0.27, p = 0.7939]. (D) Consumption of the valued flavor and the devalued during a 10-minute probe test following the prefeed. Consumption of the devalued flavor was significantly lower than the valued flavor [t(15)=4.48, p = 0.0004], indicating successful devaluation. (E) The calculated devaluation index for each subject. Devaluation index (devalued consumption) / (devalued consumption + valued consumption), scores closer to 0.5 (red dashed line) indicate insensitivity to devaluation and scores closer to 0.0 indicated sensitivity to devaluation. See Supplementary Table S1 for complete analyses. All data are presented as mean ± SEM. [file Image_1.jpeg]

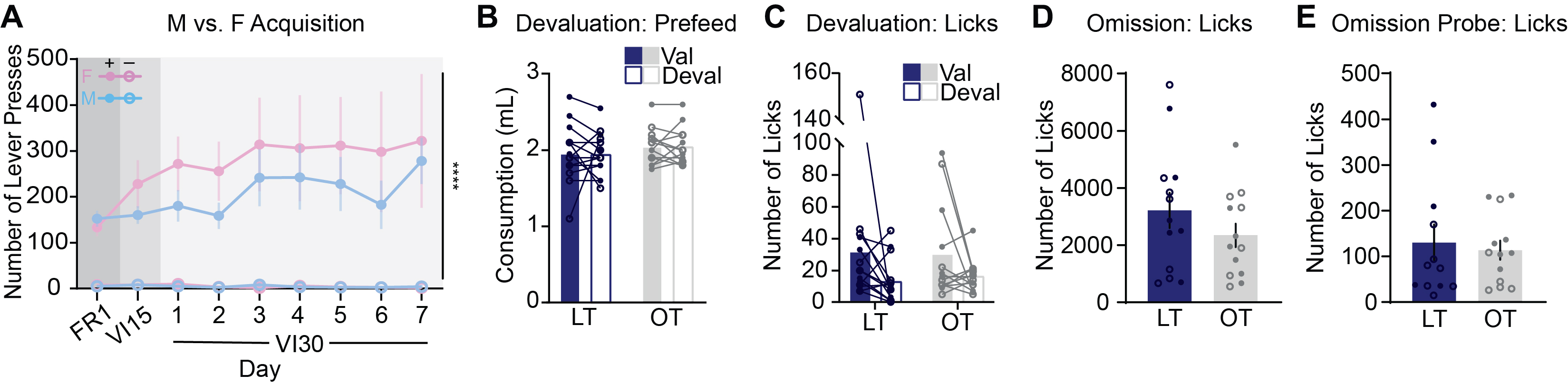

Supplement: SUPPLEMENTARY FIGURE S2 — Sex comparison, prefeed consumption, and lick data across tests. (A) Number of lever presses over training paradigms, comparing males (blue) and females (pink), as well as active (filled dot) and inactive (unfilled dot) lever presses. Lever pressing significantly increased across days, with a robust difference observed between the active and inactive lever. Males and females exhibited no significant difference in overall press rate (n = 27: 14M, 13F; mixed effects, three-way ANOVA, lever effect: F1,25 = 153.9, p < 0.0001, sex effect: F1,60 = 1.974, p = 0.1652, day x lever interaction: F1.26,9.44 = 5.274, p = 0.0401). (B) Consumption of the valued (filled bar) and the devalued (unfilled bar) flavor during the pre-feeding period did not differ between groups. (C) Number of licks during the valued and devalued test days did not differ between groups. (D) Total number of licks during the omission test in the limited-trained and overtrained groups did not differ. (E) Total number of licks during brief 10-minute omission probe test did not differ between groups. See Supplementary Table S1 for complete analyses. All data are presented as mean ± SEM. [file Image_2.jpeg]

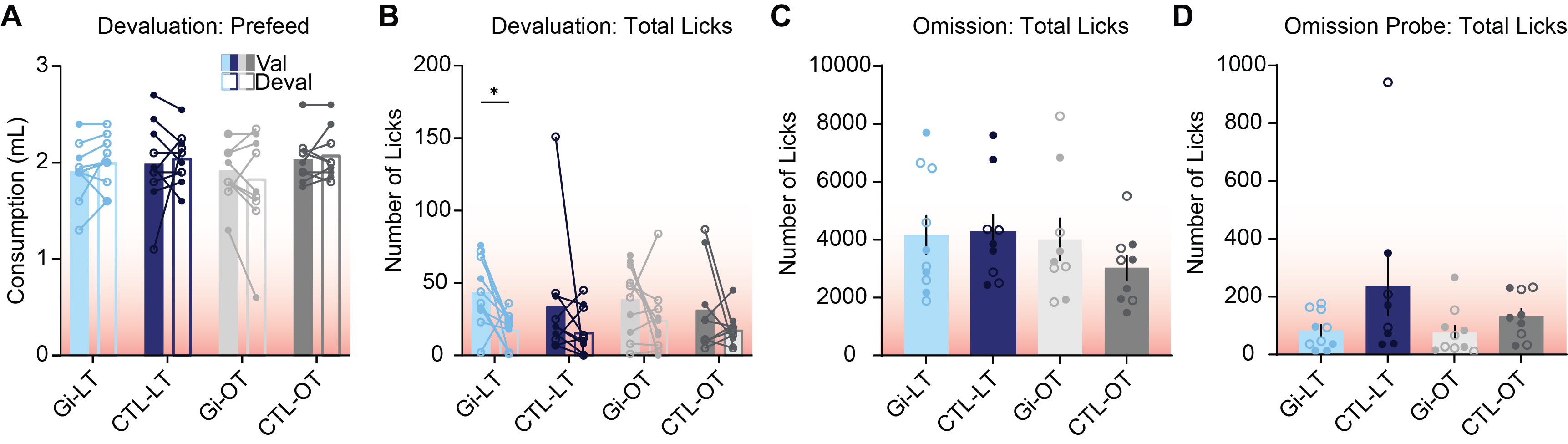

Supplement: SUPPLEMENTARY FIGURE S3 — DREADD prefeed and lick data. (A) Consumption of the valued (filled bar) and devalued (unfilled bar) flavor during the prefeed period, across all groups. Limited-trained animals and overtrained animals show similar consumption patterns. (B) The total number of licks for the valued and devalued test, Gi-LT mice licked significantly less on the devalued day compared to the valued day, with no difference found for any of the other groups (n = 39: 19M, 20F; three-way ANOVA, test day effect: F1,35 = 9.110, p = 0.0047, Two-stage BKY post hoc test, Gi-LT: p = 0.0342). (C) Total number of licks during the omission test, no difference between groups. (D) Total number of licks during omission probe test, no difference between groups. See Supplementary Table S1 for complete analyses. All data are presented as mean ± SEM. [file Image_3.jpeg]
