# Supplementary material for: Goal-directed actions and habits in head-fixed mice
Source: Front Behav Neurosci. 2026 Feb 25;20:1751553. doi: 10.3389/fnbeh.2026.1751553 (PMC12975963; doi:10.3389/fnbeh.2026.1751553)
Supplement: SUPPLEMENTARY TABLE S1 — Supplementary statistical table. [file Data_Sheet_1.pdf]

Figure	Statistical test	n	*p*-value	Post test	*p*-value
2C	Three-way ANOVA	n=27; 14M, 13F			
	Lever Effect: F (1, 25) = 58.25, (η_p^2 = 0.700)		<0.0001		
	Group Effect: F (1, 25) = 0.002, (η_p^2 < 0.001)		0.9694		
	Day Effect: F (1.30, 32.44) = 3.580, (η_p^2 = 0.125)		0.0573		
	Group x Lever Interaction: F (1, 25) = 0.047, (η_p^2 = 0.002)		0.8308		
	Day x Group Interaction: F (1.30, 32.44) = 1.638, (η_p^2 = 0.062)		0.2125		
	Day x Lever Interaction: F (1.81, 45.16) = 3.723, (η_p^2 = 0.130)		0.0359		
	Day x Group x Lever Interaction: F (1.81, 45.16) = 1.534, (η_p^2 = 0.058)		0.2274		
2D	Two-way ANOVA	n=27; 14M, 13F		Valued - Devalued: LT	<0.0001
	Test Day Effect: F (1, 25) = 17.54, (η_p^2 = 0.412)		0.0003	Valued - Devalued: OT	0.6323
	Training Effect: F (1, 25) = 0.4507, (η_p^2 = 0.018)		0.5081		
	Interaction: F (1, 25) = 29.57, (η_p^2 = 0.542)		<0.0001		
2E	Unpaired t-test	n=27: 14M, 13F			
	t (25) = 4.98		<0.0001		
2F	Unpaired t-test	n=26; 13M, 13F			
	t (24) = 3.48, (*d* = 1.73)		0.0019		
2G	Unpaired t-test	n=26; 13M, 13F			
	t (24) = 4.40		0.0002		
2H	Unpaired t-test	n=26; 13M, 13F			
	t (24) = 3.55, (*d* = 1.39)		0.0016		
3D	Three-way ANOVA	n=39; 19M, 20F		Valued:CTL-LT vs. Devalued:CTL-LT	0.0006
	Test Day Effect: F (1, 35) = 19.86, (η_p^2 = 0.362)		<0.0001	Valued:CTL-OT vs. Devalued:CTL-OT	0.255
	Virus Effect: F (1, 35) = 9.750, (η_p^2 = 0.218)		0.0036	Valued:Gi-LT vs. Devalued:Gi-LT	0.0004
	Training Effect: F (1, 35) = 0.01137, (η_p^2 < 0.001)		0.9157	Valued:Gi-OT vs. Devalued:Gi-OT	0.0145
	Test Day x Virus Interaction: F (1, 35) = 3.712, (η_p^2 = 0.096)		0.0622	Devalued:CTL-OT vs. Devalued:Gi-OT	0.0004
	Test Day x Training Interaction: F (1, 35) = 9.756, (η_p^2 = 0.218)		0.0036		
	Virus x Training Interaction: F (1, 35) = 0.6827, (η_p^2 = 0.019)		0.4143		
	Test Day x Virus x Training Interaction: F (1, 35) = 3.286, (η_p^2 = 0.086)		0.0785		
3E	Two-way ANOVA	n=39; 19M, 20F		Gi:LT vs. Gi:OT	>0.9999
	Virus Effect: F (1, 35) = 10.01		0.0032	Gi:LT vs. CTL:LT	0.8544
	Training Effect: F (1, 35) = 3.955		0.0546	Gi:LT vs. CTL:OT	0.0052
	Interaction: F (1, 35) = 4.196		0.0481	Gi:OT vs. CTL:LT	0.8342
				Gi:OT vs. CTL:OT	0.0047
				CTL:LT vs. CTL:OT	0.0378
3F	Two-way ANOVA	n=38; 19M, 19F		Gi:LT vs. Gi:OT	0.9973
	Virus Effect: F (1, 34) = 20.54, (η_p^2 = 0.377)		<0.0001	Gi:LT vs. CTL:LT	0.8807
	Training Effect: F (1, 34) = 14.12, (η_p^2 = 0.293)		0.0006	Gi:LT vs. CTL:OT	<0.0001
	Interaction: F (1, 34) = 12.16, (η_p^2 = 0.263)		0.0014	Gi:OT vs. CTL:LT	0.9466
				Gi:OT vs. CTL:OT	<0.0001
				CTL:LT vs. CTL:OT	<0.0001

Figure	Statistical test	n	*p* -value	Post test	*p* -value
3G	Two-way ANOVA	n=38; 19M, 19F		Gi:LT vs. Gi:OT	>0.9999
	Virus Effect: F (1, 34) = 21.83		<0.0001	Gi:LT vs. CTL:LT	0.9758
	Training Effect: F (1, 34) = 16.04		0.0003	Gi:LT vs. CTL:OT	<0.0001
	Interaction: F (1, 34) = 16.71		0.0003	Gi:OT vs. CTL:LT	0.9647
				Gi:OT vs. CTL:OT	<0.0001
				CTL:LT vs. CTL:OT	<0.0001
3H	Two-way ANOVA	n=38; 19M, 19F		Gi:LT vs. Gi:OT	0.9968
	Virus Effect: F (1, 34) = 20.07, (η_p^2 = 0.371)		<0.0001	Gi:LT vs. CTL:LT	0.7858
	Training Effect: F (1, 34) = 11.84, (η_p^2 = 0.258)		0.0016	Gi:LT vs. CTL:OT	<0.0001
	Interaction: F (1, 34) = 9.961, (η_p^2 = 0.227)		0.0033	Gi:OT vs. CTL:LT	0.8827
				Gi:OT vs. CTL:OT	<0.0001
				CTL:LT vs. CTL:OT	0.0004
S1A	Paired t-test	n=8			
	t (7) = 0.3456		0.7398		
S1B	Paired t-test	n=8			
	t (7) = 0.3843		0.7122		
S1C	Paired t-test	n=8			
	t (7) = 0.2714		0.7939		
S1D	Paired t-test	n=8			
	t (15) = 4.477		0.0004		
S2A	Mixed-effects Model	n=27; 14M, 13F			
	Day Effect: F (2.637, 65.91) = 1.730		0.1752		
	Sex Effect: F (1, 60) = 1.974		0.1652		
	Lever Effect: F (1.000, 25.00) = 153.9		<0.0001		
	Day x Sex Interaction: F (2.637, 19.77) = 0.5012		0.6630		
	Day x Lever Interaction: F (1.259, 9.444) = 5.274		0.0401		
	Sex x Lever Interaction: F (1.000, 60.00) = 5.175		0.0265		
	Day x Sex x Lever Interaction: F (1.259, 9.444) = 1.418		0.2735		
S2B	Two-way ANOVA	n=27; 14M, 13F			
	Test Day Effect: F (1, 25) = 0.01353		0.9083		
	Training Effect: F (1, 25) = 0.9071		0.3500		
	Interaction: F (1, 25) = 0.03246		0.8585		
S2C	Two-way ANOVA	n=27; 14M, 13F		Valued - Devalued: LT	0.1341
	Test Day Effect: F (1, 25) = 5.162		0.0320	Valued - Devalued: OT	0.3542
	Training Effect: F (1, 25) = 0.02055		0.8872		
	Interaction: F (1, 25) = 0.1299		0.7216		
S2D	Unpaired t-test	n=26; 13M, 13F			
	t (24) = 1.169		0.2540		
S2E	Unpaired t-test	n=25; 12M, 13F			
	t (23) = 0.3906		0.6997		
S3A	Three-way ANOVA	n=39; 19M, 20F			
	Test Day Effect: F (1, 35) = 0.2772		0.6019		
	Virus Effect: F (1, 35) = 1.453		0.2362		
	Training Effect: F (1, 35) = 0.04326		0.8364		
	Test Day x Virus Interaction: F (1, 35) = 0.2772		0.6019		
	Test Day x Training Interaction: F (1, 35) = 1.018		0.3200		
	Virus x Training Interaction: F (1, 35) = 0.3427		0.5620		
	Test Day x Virus x Training Interaction: F (1, 35) = 0.8047		0.3758		

Figure	Statistical test	n	*p* -value	Post test	*p* -value
S3B	Three-way ANOVA	n=39; 19M, 20F		Valued:Gi-LT vs. Devalued:Gi-LT	0.0342
	Test Day Effect: F (1, 35) = 9.110		0.0047	Valued:CTL-LT vs. Devalued:CTL-LT	0.1334
	Virus Effect: F (1, 35) = 1.392		0.2461	Valued:Gi-OT vs. Devalued:Gi-OT	0.2354
	Training Effect: F (1, 35) = 0.002223		0.9627	Valued:CTL-OT vs. Devalued:CTL-OT	0.2736
	Test Day x Virus Interaction: F (1, 35) = 0.1198		0.7314		
	Test Day x Training Interaction: F (1, 35) = 0.4500		0.5067		
	Virus x Training Interaction: F (1, 35) = 0.01009		0.9206		
	Test Day x Virus x Training Interaction: F (1, 35) = 0.09723		0.7570		
S3C	Two-way ANOVA	n=37; 18M, 19F			
	Virus Effect: F (1, 33) = 0.5060		0.4819		
	Training Effect: F (1, 33) = 1.270		0.2678		
	Interaction: F (1, 33) = 0.7506		0.3925		
S3D	Two-way ANOVA	n=37; 18M, 19F		Gi:LT vs. Gi:OT	0.9997
	Virus Effect: F (1, 33) = 4.316		0.0456	Gi:LT vs. CTL:LT	0.1661
	Training Effect: F (1, 33) = 1.209		0.2795	Gi:LT vs. CTL:OT	0.8952
	Interaction: F (1, 33) = 0.9532		0.3360	Gi:OT vs. CTL:LT	0.1411
				Gi:OT vs. CTL:OT	0.8578
				CTL:LT vs. CTL:OT	0.5019
